# Comparative Clinical Trajectories Across Cannabis‐Related and Nonsubstance‐Related Psychoses

**DOI:** 10.1111/acps.70097

**Published:** 2026-04-08

**Authors:** Antti Mustonen, Solja Niemelä, Alexander Denissoff, Antti Tanskanen, Ellenor Mittendorfer‐Rutz, Markku Lähteenvuo, Jari Tiihonen, Heidi Taipale

**Affiliations:** ^1^ Faculty of Medicine and Health Technology Tampere University Tampere Finland; ^2^ Department of Clinical Neuroscience Karolinska Institutet Stockholm Sweden; ^3^ Department of Psychiatry Seinäjoki Central Hospital Seinäjoki Finland; ^4^ Department of Psychiatry, Faculty of Medicine University of Turku Turku Finland; ^5^ Mental Health and Addiction Services, Wellbeing County of South‐West Finland Turku Finland; ^6^ Department of Forensic Psychiatry, Niuvanniemi Hospital University of Eastern Finland Kuopio Finland; ^7^ Neuroscience Center, University of Helsinki Helsinki Finland; ^8^ Center for Psychiatry Research, Stockholm Region Stockholm Sweden; ^9^ School of Pharmacy University of Eastern Finland Kuopio Finland

**Keywords:** cannabis‐induced psychosis, first‐episode psychosis, hospitalization, mortality, outcome, register study, trajectory

## Abstract

**Background:**

Research indicates that first‐episode psychosis (FEP) with cannabis use disorder (CUD) carries a substantial disease burden, but more granular clinical trajectories of cannabis‐related psychoses remain unclear. This study examines the clinical trajectories of first clinically diagnosed FEP&CUD and cannabis‐induced psychosis (CIP) in comparison with FEP without substance use disorder (SUD).

**Methods:**

From the linkage of nationwide administrative and medical registers of Sweden (2006–2021), we identified 1772 individuals (84.1% men) with incident CIP, 1360 individuals (84.3% men) with FEP&CUD and matched individuals with FEP but without SUD and followed them up until 2023. We compared annual prevalence of psychiatric diagnoses before and after cohort entry, medication use, days in inpatient care, and the risks of hospitalization for psychosis and death.

**Results:**

Mean age at first diagnosis was 26.6 years (SD 8.3) for incident CIP and 26.9 years (SD 8.4) for FEP&CUD and FEP without SUD. Individuals in the FEP without SUD cohort were more likely to have a record of schizophrenia diagnosis compared to the FEP&CUD and CIP cohorts during the first (14.41%, 6.25%, 2.37%) and second year (10.88%, 7.13%, 3.27%) after cohort entry. However, FEP&CUD and CIP cohorts had a more pronounced burden of inpatient treatment as well as elevated risks of hospitalization for psychosis (aHR = 1.66; 95% CI 1.49–1.86 and aHR = 1.47; 1.32–1.64) and death (aHR = 2.02; 1.44–2.82 and aHR = 1.80; 1.30–2.50) compared to individuals with FEP without SUD.

**Conclusions:**

Cannabis‐related psychoses, that is, FEP&CUD and CIP, were associated with poor outcomes, including high risk of hospitalization for psychosis and increased mortality, underscoring the need for targeted interventions. Moreover, the overlapping clinical trajectories suggest that cannabis‐related psychoses may not represent clinically distinct entities but instead lie on a continuum.

## Abstract (highlights)

**Significant Outcomes:**

- Cannabis‐related psychoses (FEP&CUD and CIP) were associated with a higher risk of hospitalization for psychosis and higher all‐cause mortality compared to FEP without SUD, underscoring the need for targeted interventions.
- Overlapping clinical trajectories suggest that cannabis‐related psychoses may lie on a psychosis continuum rather than representing clinically distinct entities.

**Limitations:**

- We did not have data on continued cannabis use beyond register information, which is relevant because ongoing use is associated with more frequent relapses and a higher burden of inpatient care in psychosis.
- Misclassification due to variability in clinical practices as well as incomplete outpatient data prior to 2001 may have affected case identification.

## 1 Introduction

Cannabis use and cannabis use disorder (CUD) significantly worsen prognosis of psychotic disorders. Comorbidity between psychotic disorders and CUD is common, with rates reaching up to 36% in first‐episode psychosis (FEP) samples [1]. Continued cannabis use exacerbates the course of psychotic disorders, leading to higher rates of nonadherence to antipsychotic medications [2, 3], increased relapse risk, frequent and prolonged inpatient treatments [4, 5, 6, 7], treatment resistance [8], and elevated mortality [9]. These findings highlight the substantial burden of disease and mortality linked to cannabis use in psychotic disorders.

Cannabis‐induced psychosis (CIP) is recognized in both ICD‐10 and DSM‐5 as a condition where psychotic symptoms persist beyond intoxication and withdrawal [10, 11]. Although diagnostic systems do not define strict time criteria, symptoms are generally expected to last at least 48 h and resolve within one to 6 months [10, 11, 12]. However, evidence suggests that individuals with CIP are often subsequently diagnosed with primary psychoses [13, 14, 15, 16, 17, 18] with meta‐analytical data indicating approximately one‐third of individuals with CIP eventually develop schizophrenia [19].

A recent expert review raised concerns about the construct validity of substance‐induced psychosis (SIP) diagnoses. Bramness and colleagues highlighted concerns that SIP diagnoses may overemphasize the role of substance use as a mechanistic cause for the psychotic state [20]. Since SIPs are often considered self‐limiting, this perception influences how frequently individuals receive evidence‐based treatments, contributing to undertreatment and inequality [20, 21].

Comparative studies between SIP and FEP yield inconsistent results. A systematic review did not find many consistent differences in psychopathology, but reported weaker family history of psychosis, greater insight, and fewer positive and negative symptoms in SIP compared to primary psychosis with substance use disorder (SUD) [22]. More recent studies have not been able to replicate these findings, reporting no significant differences between SIP and FEP with respect to sociodemographic factors or clinical symptom severity [21, 23], antipsychotic use [21], frequency or duration of subsequent inpatient treatments [21], or relapse and recovery rates [23, 24]. The few studies specifically on CIP suggest elevated rates of anxiety and depression in CIP [25] while others report lower levels of depression and anxiety but higher rates of SUD in CIP compared to FEP [26]. Still, other studies have found no significant differences in psychiatric symptoms between the two groups [27].

There is a lack of robust long‐term data specifically comparing the outcomes of CIP and FEP and the impact of CUD to the prognosis of FEP. Nationwide register‐based datasets allow for the analysis of larger, more representative populations using real‐world clinical data. Our previous research utilizing Swedish national registers has shown that both FEP&CUD [28] and CIP [29] are associated with high relapse rates and that antipsychotic medications are effective in preventing relapse in both groups [28, 29].

### 1.1 Aims of the Study

The present study aims to extend our previous work by characterizing individuals with first clinically diagnosed FEP&CUD, CIP, and FEP without a SUD, focusing on demographic features, psychiatric comorbidities, medication use, and the risks of hospitalization for psychosis and death. We hypothesized that cannabis‐related psychoses would be associated with a more adverse clinical trajectory after first presentation compared with FEP without SUD. Identifying distinct clinical profiles and outcomes may support the development of more targeted treatment strategies and contribute to precision medicine approaches for these patient groups. Additionally, we hypothesized that FEP & CUD and CIP share broadly similar clinical trajectories and may represent related manifestations rather than fully distinct clinical entities.

## 2 Materials and Methods

### 2.1 Study Population

This study utilizes data from several comprehensive Swedish national registers, encompassing all individuals residing in Sweden. Each resident is assigned a unique personal identification number, which allows for the linkage of various registers after de‐identification. The registers include the National Patient Register (NPR), Micro Data for Analyses of Social Insurance (MiDAS), Cause of Death Register (CDR), Prescribed Drug Register (PDR), and the Longitudinal Integration Database for Health Insurance (LISA).

The NPR provides data on inpatient and specialized outpatient care, while MiDAS contains information on sickness absence and disability pensions, specifically periods during which individuals received sickness benefits due to health‐related work incapacity. From the NPR and MiDAS registers, we formed three mutually exclusive cohorts that included all individuals aged 16–64 years who were first diagnosed with: (1) nonaffective psychotic disorder as a proxy for FEP (International Classification of Diseases, 10th Revision; ICD‐10 codes F20–F29) with co‐occurring CUD, defined as any cannabis‐use–related diagnosis (ICD‐10 codes F12.0–F12.9) recorded within 2 weeks before or after the incident FEP diagnosis (FEP&CUD); (2) CIP (ICD‐10 code F12.5); and (3) nonaffective psychotic disorder as a proxy for FEP (ICD‐10 codes F20–F29) without SUD (ICD‐10 codes F10‐F19), defined as having no diagnosed SUD at any time before or within 1 year after the index FEP diagnosis and matched with the FEP&CUD cohort by age (±2 years window), sex, and year of cohort entry (FEP without SUD). If an individual had both CIP and FEP&CUD diagnosis within 2 weeks, they were assigned to the FEP&CUD cohort. Inclusion period to the cohorts was between January 2006 and December 2021. Individuals in these cohorts were selected based on the absence of prior diagnoses (since 1997) of SIP (F1x.5), schizophrenia‐spectrum disorders (F20–F29), or bipolar disorder (F30–F31), ensuring the inclusion of only incident psychosis cases. No other exclusion criteria were applied in this study.

### 2.2 Key Variables and Covariates

Sociodemographic data, including age, sex, educational level, country of birth, income and occupational information, were obtained from LISA. Data on number and length of inpatient treatments for psychosis in addition to record of diagnoses of schizophrenia (F20), bipolar disorder (F30–F31), depression (F32–F33), anxiety disorder (F40–F43), alcohol use disorder (F10), opioid use disorder (F11), CUD (F12), sedative use disorders (F13), stimulant use disorder (F15), polysubstance use disorder (F19), overdoses (T36–T50), and suicide attempts (X60–X84, Y10–Y34) in registers were obtained from the NPR. Medication data were gathered from the PDR from July 2005 to December 2023 and were categorized into based on Anatomical Therapeutic Chemical (ATC) classification code [30] as antipsychotics (N05A, excluding N05AN01 [lithium]), medications for SUDs (N07BB, N07BC), medications for attention‐deficit hyperactivity disorder (N06BA), mood stabilizers (N03AF01, N03AG01, N03AX09, N05AN01), antidepressants (N06A) and benzodiazepines and related drugs (N05BA, N05CD, N05CF). Medication data were modelled into medication use periods with the PRE2DUP (from prescription drug purchases to drug use periods) method described elsewhere [31].

### 2.3 Outcomes

Information on dates of death was collected from the Registry for Causes of Death. Information on hospitalization for psychosis was collected from NPR and was defined as an inpatient admission with ICD‐10 F20–F29 or any SIP (F1x.5) diagnosis.

### 2.4 Statistical Methods

We calculated annual prevalence rates of various psychiatric disorders for the intervals spanning one to 5 years prior to and one to 2 years after the FEP&CUD, CIP, and FEP without SUD diagnoses. Cross‐tabulation with *χ* ^2^ or Fisher's exact test, as appropriate, was used to assess whether annual prevalence of records of schizophrenia, bipolar disorder, depression, anxiety disorder, alcohol use disorder, CUD, opioid use disorder, sedative use disorders, stimulant use disorder, polysubstance use disorder, overdose, and suicide attempt diagnoses in registers were different between FEP&CUD, CIP, and FEP without SUD cohorts. Prevalence curves for these diagnoses were constructed to illustrate the distribution of these diagnoses over time by the exposure group. Furthermore, differences in highest education received, sex, income past year, disability pension at cohort entry, having received sickness absence benefits during the past year, age at first diagnosis, days spent in hospital, and medication use were compared.

We used Cox‐regression analysis with hazard ratios (HRs) and 95% confidence intervals (CI) to assess the risk of death and psychosis between FEP&CUD, CIP and FEP without SUD (reference) cohorts. Furthermore, we conducted supplementary analysis comparing outcomes between FEP&CUD and CIP (see Tables S1 and S2). These models were adjusted for sex, income past year, disability pension at cohort entry, having received sickness absence benefits during the past year, age at first diagnosis and calendar year of cohort entry. Cumulative incidence curves for these outcomes were also generated. Patients were followed up from first psychosis diagnosis until emigration (LISA), death (CDR), or end of the data linkage (December 2023), which ever occurred first. Statistical significance was considered at > 0.05. Statistical analyses were performed using SAS version 9.4 for Windows (SAS Institute Inc., Cary, NC, USA; https://www.sas.com/fi_fi/software/iml‐sas9.html). Prevalence and cumulative incidence curves were created using R version 4.1.1 for Windows (R Foundation for Statistical Computing, Vienna, Austria; https://www.R‐project.org/).

## 3 Results

The study cohorts comprised 1360 individuals with FEP&CUD (84.3% men), 1772 individuals with incident CIP (84.1% men), and 1360 with FEP without SUD (84.3% men). The mean age at first diagnosis was 26.9 years (SD 8.4) for FEP&CUD, 26.6 years (SD 8.3) for CIP, and 26.9 years (SD 8.4) for FEP&SUD. No significant differences were observed between the cohorts in terms of sex or age at first diagnosis. For other comparisons see Table 1.

**TABLE 1:** Comparison of demographic and clinical correlates between individuals with first‐episode psychosis with cannabis use disorder (FEP&CUD; *n* = 1360), incidentcannabis‐induced psychosis (CIP; *n* = 1772) and first‐episode psychosis without substance use disorder (FEP without SUD; *n* = 1360).

Exposure variable	FEP and CUD		CIP		FEP without SUD		*p*
	Frequency (*n*)	Percent (%)	Frequency (*n*)	Percent (%)	Frequency (*n*)	Percent (%)	
Age (years)							
16–19	161	11.84	232	13.09	160	11.76	0.534
20–24	523	38.46	681	38.43	500	36.76	
25–29	325	23.90	399	22.52	347	25.51	
≥ 30	351	25.81	460	25.96	353	25.96	
Sex							
Female	214	15.74	282	15.91	214	15.74	0.987
Male	1146	84.26	1490	84.09	1146	84.26	
Born in Sweden							
No	403	29.63	501	28.27	445	32.72	**0.025**
Yes	957	70.37	1271	71.73	915	67.28	
Education							
Elementary	658	48.38	830	46.83	575	42.23	**< 0.001**
High School	569	41.84	752	42.44	545	40.07	
University	133	9.78	190	10.72	240	17.65	
Income from work							
No	610	44.85	777	43.85	659	48.46	**0.031**
Yes	750	55.15	995	56.15	701	51.54	
Sickness absence previous year							
No	1070	78.68	1495	84.37	1082	79.56	**< 0.0001**
1–90 days	204	15.00	181	10.21	215	15.81	
≥ 90 days	86	6.32	96	5.42	63	4.63	
Disability pension at cohort entry							
No	1211	89.04	1649	93.06	1136	83.53	**< 0.0001**
Yes	149	10.96	123	6.94	224	16.47	
Hospitalization during the first year after cohort entry							
No	865	63.60	1187	66.99	1032	75.89	**< 0.0001**
Yes	495	36.40	585	33.01	328	24.11	
Hospitalization during the second year after cohort entry							
No	1087	79.93	1484	83.75	1204	88.53	**< 0.0001**
Yes	273	20.07	288	16.25	156	11.47	
Days in hospital care during the first year after cohort entry							
No	866	63.68	1187	66.99	1033	75.96	**< 0.0001**
1–6	35	2.57	58	3.27	14	1.03	
7 or more	459	33.75	527	29.74	313	23.01	
Days in hospital care during the second year after cohort entry							
No	1088	80.00	1458	83.80	1206	88.68	**< 0.0001**
1–6	26	1.91	30	1.69	9	0.66	
7 or more	246	18.09	257	14.50	145	10.66	

*Note:* Statistically significant associations in bold.

**FIGURE 1:**
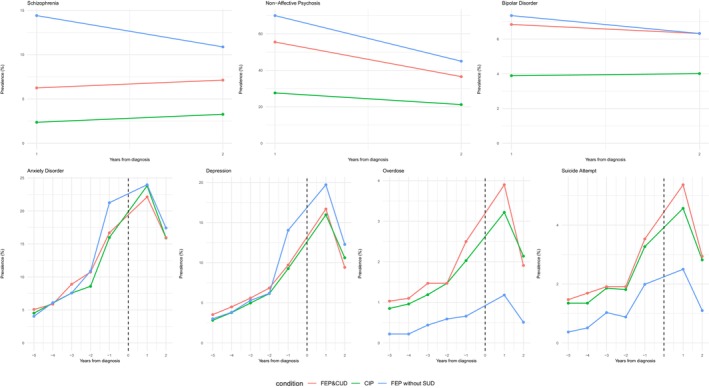
The annual prevalence of recorded psychiatric diagnoses in the registers from 1 to 5 years before cohort entry to 1–2 years after cohort entry among individuals with first‐episode psychosis and cannabis use disorder (FEP&CUD), cannabis‐induced psychosis (CIP), and first‐episode psychosis without substance use disorder (FEP without SUD).

The annual prevalence of recorded anxiety disorder diagnoses in the registers showed variable trends across the three cohorts. In contrast, depression was generally more common in the FEP without SUD cohort from 1 year before cohort entry until one and 2 years after cohort entry. Annual records of diagnoses related to overdoses and suicide attempts were consistently less common in the FEP without SUD cohort from one to 5 years prior to cohort entry through one to 2 years after cohort entry. In comparison, the prevalence of all these diagnoses was largely similar between the FEP&CUD and CIP cohorts. Records of schizophrenia diagnoses were more frequently observed in the registers for the FEP without SUD cohort than for the FEP&CUD or CIP cohorts during the first (14.41%, 6.25%, 2.37%) and second year (10.88%, 7.13%, 3.27%) after cohort entry as were nonaffective psychosis diagnoses (70.0%, 55.51%, 27.60% and 45.00%, 36.54%, 21.22%). Diagnoses of bipolar disorder were recorded more frequently in the registers for the FEP without SUD and FEP&CUD cohorts than for the CIP cohort during the first and second years after cohort entry (7.35%, 6.84%, 3.89% and 6.32%, 6.32% and 4.01%). For illustration, see Figure 1.

**FIGURE 2:**
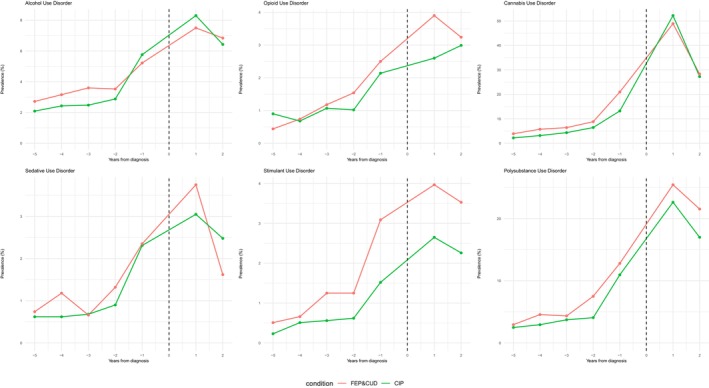
The annual prevalence of recorded substance use disorder diagnoses in the registers from 1 to 5 years before cohort entry to 1–2 years after cohort entry among individuals with first‐episode psychosis and cannabis use disorder (FEP&CUD), cannabis‐induced psychosis (CIP), and first‐episode psychosis without substance use disorder (FEP without SUD).

In terms of the annual prevalence of recorded SUDs between the FEP&CUD and CIP cohorts, alcohol use disorder, opioid use disorder, and sedative use disorder was largely similar between the cohorts during 1–5 years prior to and the first and second years after cohort entry. Records of CUD diagnoses were more common in the FEP&CUD cohort during the one to 5 years preceding cohort entry but were comparable between the cohorts during the first and second years after cohort entry. Polysubstance and stimulant use disorders diagnoses demonstrated variable trends over time but were generally more common in the FEP&CUD group. For illustration, see Figure 2.

Individuals in the FEP without SUD cohort were more likely to have used any antipsychotic 30 days after cohort entry (57.06%), whereas the rate was similar between FEP&CUD (51.8%) and CIP (48.16%) cohorts. Use of long‐acting injectable antipsychotics (LAIs) was infrequent overall, but more common in the FEP&CUD (2.72%) and FEP without SUD (2.79%) cohorts compared to the CIP cohort (0.34%) 30 days after cohort entry. The use of benzodiazepines and mood stabilizers was more common in both FEP cohorts, whereas the use of ADHD medications was comparable across all three cohorts 30 days after cohort entry. Antidepressants were more frequently used in the FEP without SUD (29.41%) cohort than the FEP&CUD (21.95%) and CIP cohorts (21.25%) 30 days after cohort entry. For all the comparisons, including the medication use 180 days before cohort entry, see Table 2.

**TABLE 2:** Comparison of medication use 180 days before and 30 days after cohort entry between individuals with first‐episode psychosis with cannabis use disorder (FEP&CUD; *n* = 1360), incident cannabis‐induced psychosis (CIP; *n* = 1772) and first‐episode psychosis without substance use disorder (FEP without SUD; *n* = 1360).

Exposure variable	FEP and CUD		CIP		FEP without SUD		*p*
	Frequency (*n*)	Percent (%)	Frequency (*n*)	Percent (%)	Frequency (*n*)	Percent (%)	
30 days after cohort entry							
Any antipsychotic							
No	705	51.84	973	54.91	584	42.94	**< 0.0001**
Yes	655	48.16	799	45.09	776	57.06	
Long‐acting injectable antipsychotics							
No	1323	97.28	1766	99.66	1322	97.21	**< 0.0001**
Yes	37	2.72	6	0.34	38	2.79	
Any antidepressant							
No	1071	78.75	1383	78.05	960	70.59	**< 0.0001**
Yes	289	21.25	389	21.95	400	29.41	
ADHD medications							
No	1309	96.25	1714	96.73	1316	96.76	0.7030
Yes	51	3.75	58	3.27	44	3.24	
Benzodiazepines							
No	1246	91.62	1666	94.02	1191	87.57	**< 0.0001**
Yes	114	8.38	106	5.98	169	12.43	
SUD medications							
No	1337	98.31	1737	98.02	1358	NA	< **0.0001**
Yes	23	1.69	35	1.98	< 5	NA	
Mood stabilizers							
No	1284	94.41	1714	96.73	1267	93.16	**< 0.0001**
Yes	76	5.59	58	3.27	93	6.84	
180 days before cohort entry							
Any antidepressant							
No	897	65.96	1181	66.65	802	58.97	**< 0.0001**
Yes	463	34.04	591	33.35	558	41.03	
ADHD medications							
No	1287	94.63	1686	95.15	1304	95.88	0.307
Yes	73	5.37	86	4.85	56	4.12	
Benzodiazepines							
No	1188	87.35	1593	89.90	1118	82.21	**< 0.0001**
Yes	172	12.65	179	10.10	242	17.79	
SUD medications							
No	1326	97.50	1723	97.23	1355	99.63	**< 0.0001**
Yes	34	2.50	49	2.77	5	0.37	
Mood stabilizers							
No	1232	90.59	1656	93.45	1231	90.51	**0.0026**
Yes	76	9.41	116	6.55	129	9.49	

*Note:* Statistically significant associations in bold.

Abbreviations: ADHD, attention deficit hyperactivity disorder; SUD, substance use disorder.

All the cohorts experienced a substantial burden of inpatient treatment for psychosis. During the first year after cohort entry, hospitalization rates due to psychosis were higher in the FEP&CUD cohort (36.40%) and CIP cohort (33.01%) compared to the FEP without SUD cohort (24.11%). In the second year, hospitalization rates declined in all cohorts but remained higher in the FEP&CUD (20.07%) and CIP (16.25%) compared to the FEP without SUD cohort (11.47%). Furthermore, the duration of psychosis‐related hospitalizations was generally longer in the FEP&CUD and CIP cohorts during the first and second years after cohort entry compared to the FEP without SUD cohort (see Table 1 for detailed data).

**FIGURE 3:**
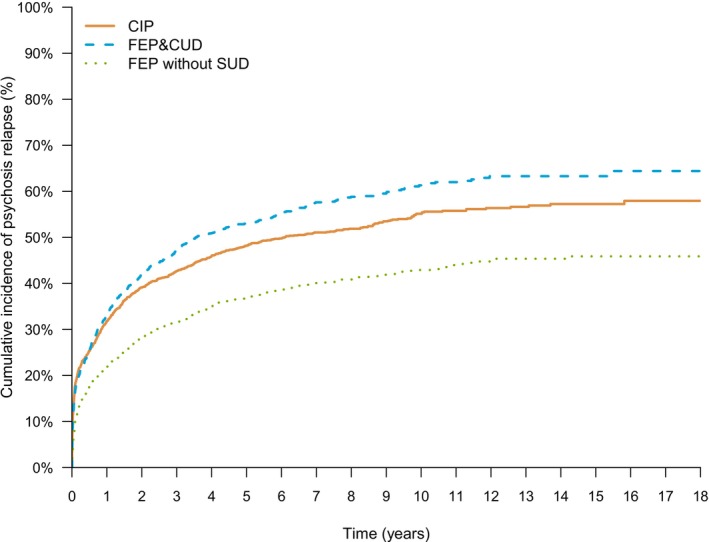
Risk of hospitalization for psychosis among individuals with first‐episode psychosis and cannabis use disorder (FEP&CUD), cannabis‐induced psychosis (CIP), and first‐episode psychosis without substance use disorder (FEP without SUD).

During the follow‐up, 762 individuals (56.03%) in the FEP&CUD cohort, 914 in the CIP cohort (51.58%), and 549 (40.4%) in the FEP without SUD cohort were hospitalized due to psychotic relapse. Individuals in the FEP&CUD (aHR = 1.66; 95% CI 1.49–1.86) and CIP cohorts (aHR = 1.47; 95% CI 1.32–1.64) had an increased risk of hospitalization due to psychotic relapse compared to those in the FEP without SUD cohort. Cumulative incidence curves for psychosis relapse are presented in Figure 3. Furthermore, individuals in the FEP&CUD cohort had an increased risk of hospitalization due to psychotic relapse compared to individuals in the CIP cohort (aHR = 1.14; 95% CI 1.03–1.25). Covariates and HRs for adjusted model (FEP&CUD vs. CIP) are presented in Table S1.

**FIGURE 4:**
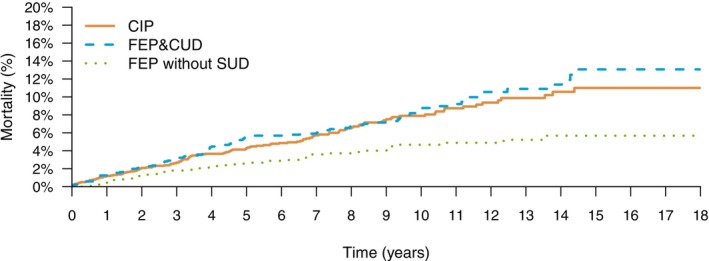
Risk of death among individuals with first‐episode psychosis and cannabis use disorder (FEP&CUD), cannabis‐induced psychosis (CIP), and first‐episode psychosis without substance use disorder (FEP without SUD).

During the follow‐up, 103 individuals (7.57%) in the FEP&CUD cohort, 123 in the CIP cohort (6.94%), and 52 (3.82%) in the FEP without SUD cohort died. Individuals in the FEP&CUD (aHR = 2.02; 95% CI 1.44–2.82) and CIP cohorts (aHR = 1.80; 95% CI 1.30–2.50) had an increased risk of death compared to individuals in the FEP without SUD cohort. Cumulative incidence curves for mortality are presented in Figure 4. There was no statistically significant difference in risk of death between the FEP&CUD and CIP cohorts (aHR = 1.10; 95% CI 0.84–1.43). Covariates and HRs for adjusted model (FEP&CUD vs. CIP) are presented in Table S2.

## 4 Discussion

First‐episode psychosis with co‐occurring cannabis use disorder (FEP&CUD) was associated with worse clinical trajectory compared to FEP without SUD and CIP. Individuals in the FEP&CUD cohort experienced a greater burden of inpatient care, including higher rates of hospitalizations due to psychotic relapse and longer durations of inpatient treatment. These findings highlight the importance of clinicians recognizing the impact of co‐occurring CUD on the prognosis of FEP at the time of first service presentation to optimize clinical outcomes. However, FEP&CUD and incident CIP cohorts shared similar demographic profiles, clinical comorbidities, and mortality risk, underscoring the need for comprehensive clinical approaches to address the disease burden associated with both conditions. These similarities also suggest that cannabis‐related psychoses may exist along a continuum rather than representing fully distinct diagnostic entities.

This is the first study to leverage nationwide datasets to examine the clinical and demographic correlates of FEP&CUD, CIP and FEP without SUD. Our findings have important clinical implications, shedding light on the similarities and distinctions between cannabis‐related psychotic conditions and their prognoses. As this is the first study utilizing nationwide registry data, direct comparisons with previous studies based on clinical samples are limited. Consistent with prior research, we observed a predominance of males in both groups [21, 26, 27, 32]. Some earlier clinical studies have reported a younger age at admission for psychosis in FEP with substance use compared to SIP [21, 32], that we were not able to replicate in this population‐based sample. This discrepancy may be attributable to our larger dataset including all visits to specialized healthcare with inclusion of both inpatient and outpatient visits, as well as our focus on recorded CUD diagnosis rather than substance or cannabis use.

Hospitalization for psychosis during the first year after cohort entry was more common in the FEP &CUD (36%) and CIP cohorts (33%) than in the FEP without SUD cohort (24%) (Table 2), and most hospitalizations in all cohorts occurred within the first three years (Figure 4). The FEP&CUD cohort received more intensive inpatient care, characterized by longer hospital stays and a higher risk of hospitalization for psychosis compared to the CIP and FEP without SUD cohorts. Mortality was high and comparable between the FEP&CUD and CIP cohorts, but higher than in the FEP without SUD cohort, suggesting a worse overall prognosis in cannabis‐related psychoses.

Notably, individuals in the FEP without SUD cohort more often received a subsequent schizophrenia diagnosis within 2 years of the incident psychosis (14.41%–10.88%). In contrast, such diagnosis remained uncommon in cannabis‐related psychoses, recorded in only 6%–7% of FEP&CUD and 2%–3% of CIP cases during the first and second years after cohort entry. This suggests potential underdiagnosis and delay of schizophrenia diagnosis in cannabis‐related populations despite evidence of worse clinical trajectory. Clinicians may initially attribute symptoms to cannabis use, particularly when intoxication or heavy use is evident, potentially delaying the assignment of a schizophrenia diagnosis even when the underlying trajectory aligns with primary psychotic disorders. Our findings emphasize earlier identification and sustained treatment of cannabis‐related psychotic disorders to mitigate the clinical trajectories of these individuals.

Individuals in FEP without SUD cohort had higher rates of any antipsychotic use 30 days after cohort entry (57%) compared to the FEP&CUD and CIP cohorts where approximately 48% and 45% received antipsychotic treatment. This suggests that, in the context of pharmacotherapy, undertreatment of cannabis‐related psychoses may occur after the first service presentation despite their poorer clinical trajectory. Despite the lack of guidelines on pharmacotherapy for SIP, individuals experiencing FEP in the context of problematic cannabis use received comparable antipsychotic treatment during the initial phase of care, regardless of whether their psychosis is classified as substance‐induced (F1x.5) or primary (F2x). However, the FEP&CUD cohort was more likely to receive LAIs within 30 days, which may reflect a clinical perception that SIP often resolves with abstinence. Additionally, individuals with FEP&CUD were more likely to have records of comorbid polysubstance use and stimulant use disorder diagnoses and worse occupational functioning, which may be associated with more frequent nonadherence and greater clinical severity, potentially contributing to more frequent use of LAIs as well as higher risk of hospitalization for psychosis. In general, use of LAIs within 30 days after cohort entry was strikingly low in all cohorts (0.34%–2.79%). Our recent studies have demonstrated that LAIs, alongside clozapine, are associated with favorable real‐world effectiveness in relapse prevention for both CIP and FEP&CUD [28, 29]. Given the findings of this study regarding more severe clinical trajectories in cannabis‐related psychoses, prescribers should consider increasing the use of LAIs to improve treatment adherence and outcomes in these populations.

The construct validity of SIP has been recently challenged [20]. Evidence suggests similar rates of subsequent schizophrenia diagnoses after a first episode of brief, atypical, or not otherwise specified psychosis compared to CIP [19], as well as the similar severity of psychotic symptoms, relapse risk and remission rates [23, 24] and treatment response observed in FEP&CUD [28, 29]. This complexity calls for a nuanced reconsideration of the conceptual distinctions between primary psychosis and SIP and especially with CIP. Rather than treating these conditions as discrete diagnostic entities, it may be more appropriate to conceptualize them along a continuum of psychotic symptomatology, reflecting the considerable overlap and heterogeneity in clinical presentation. Within this context, we argue that CIP should be seen as a subtype of primary psychosis in individuals with cannabis use, rather than a disorder solely secondary to substance use.

Our study has notable strengths and some important limitations. To our knowledge, this is the first study to examine comparative clinical trajectories of FEP&CUD, CIP and FEP without SUD using nationwide register‐based data with a large sample size. The comprehensive coverage of the Swedish national registers, which include all residents, enables accurate identification of first‐episode cases with long‐term follow‐up. However, certain limitations must be acknowledged. We lacked objective information on continued cannabis use beyond what was recorded in the registers, which is relevant as ongoing use is associated with more frequent relapses and higher burden from inpatient care in psychosis [6]. Nevertheless, we did not observe differential rates of CUD diagnoses between CIP and FEP&CUD cohorts after cohort entry, suggesting this limitation is unlikely to introduce major bias. The reliance on register‐based diagnoses may introduce misclassification due to variability in clinical practices, and incomplete outpatient data prior to 2001 may have affected case identification. Furthermore, to our knowledge, the validity of SIP diagnoses within population‐based registers has not been rigorously established. Additionally, the registers do not capture detailed clinical information such as symptom severity or psychosocial factors. Finally, our findings may not be fully generalizable to populations outside Sweden. Thus, subsequent clinical studies in diverse populations and treatment settings are warranted to examine the clinical trajectories of cannabis‐related psychoses. Despite these limitations, our study provides valuable evidence to inform future clinical practice and research on cannabis‐related psychotic disorders.

## 5 Conclusions

FEP&CUD and CIP were associated with poorer outcomes compared to FEP without SUD, including higher risk of hospitalization for psychosis, higher all‐cause mortality, and greater inpatient treatment burden, highlighting the need for targeted interventions for cannabis‐related psychosis. Although FEP&CUD followed a worse clinical course than CIP, FEP&CUD and CIP showed marked overlap in clinical and demographic characteristics. This overlap suggests that CIP may not be clinically distinct from FEP&CUD and that cannabis‐related psychoses may lie on a continuum. These results support comprehensive management approaches to mitigate the burden of disease in cannabis‐related psychotic conditions.

## Author Contributions

A.M. and H.T. developed conception and design of the work. H.T. and A.T. performed data analysis. A.M., H.T., J.T., A.T., E.M.‐R., A.D., M.L., and S.N. made the interpretation of the results and A.M. wrote the first manuscript draft. A.M., H.T., J.T., A.T., E.M.‐R., M.L., A.D., and S.N. supervised conception and design of the work and provided critical revision of the article. All authors approved the final version of the manuscript.

## Funding

This study was funded by the Swedish Research Council (2024‐03340), Juho Vainio Foundation, State funding for university‐level health research, Tampere University Hospital, and the Finnish Foundation for Alcohol Studies. We utilized data from the REWHARD consortium supported by the Swedish Research Council (grant number 2021‐00154). The funders of the study had no role in study design, data collection, data analysis, data interpretation, or writing of the report.

## Ethics Statement

The project was approved by the Regional Ethical Review Board, Karolinska Institutet, Stockholm, Sweden (Dnr: 2007/762‐31 and Dnr 2024‐08708‐02). According to current Swedish legislation, the use of registry data for research purposes does not require informed consent from the individuals included in these registries.

## Conflicts of Interest

A.M. has received funding from Juho Vainio Foundation, The Wellbeing Services County of South Ostrobothnia, State funding for university‐level health research, Tampere University Hospital, The Wellbeing Services County of South Ostrobothnia, and The Finnish Foundation for Alcohol Studies. A.M. has received lecture fees from Recordati, Lundbeck, and Otsuka; advisory board compensation from Recordati; and travel and congress support from Recordati. A.D. has received funding from Juho Vainio Foundation, Yrjö Jahnsson Foundation, and personal fees from Finnish State Research Funding (ERVA). H.T. is funded by Sigrid Juselius Foundation. J.T., E.M.‐R., H.T., and A.T. have participated in research projects funded by grants from Janssen‐Cilag to their employing institution. M.L. has received honoraria from Johnson & Johnson, Lundbeck, Orion Pharma, Otsuka, Recordati, and Teva. S.N. reports personal fees from dne Pharma, Otsuka, Lundbeck, Recordati, and Shire‐Takeda. H.T. reports personal fees from Gedeon Richter, Janssen‐Cilag, Lundbeck, and Otsuka. J.T. has served as a consultant for Healthcare Global Village, HLS Therapeutics, Janssen, Orion, Teva, and WebMD Global and has received honoraria from Janssen‐Cilag, Lundbeck, and Otsuka.

## Supporting information


**Data S1:** Supporting Information Table S1 and Table S2. Risk of death and hospitalization due to psychotic relapse, and associated baseline variables in a sample of individuals with first‐episode psychosis and cannabis use disorder (FEP&CUD, *N* = 1360) and cannabis‐induced psychosis (CIP, *N* = 1772).

## Data Availability

The data used in this study cannot be made publicly available due to privacy regulations. According to the General Data Protection Regulation, the Swedish law SFS 2018:218, the Swedish Data Protection Act, the Swedish Ethical Review Act, and the Public Access to Information and Secrecy Act, these types of sensitive data can only be made available for specific purposes, including research, that meets the criteria for access to this sort of sensitive and confidential data as determined by a legal review. Readers may contact Professor Ellenor Mittendorfer‐Rutz (ellenor.mittendorfer-rutz@ki.se) regarding the data.
