# Supplementary material for: Comparative Clinical Trajectories Across Cannabis‐Related and Nonsubstance‐Related Psychoses
Source: Acta Psychiatr Scand. 2026 Apr 8;154(2):142–52. doi: 10.1111/acps.70097 (PMC13327216; doi:10.1111/acps.70097)
Supplement: Supplementary file 1 — Data S1: Supporting Information Table S1 and Table S2. Risk of death and hospitalization due to psychotic relapse, and associated baseline variables in a sample of individuals with first‐episode psychosis and cannabis use disorder (FEP&CUD, N = 1360) and cannabis‐induced psychosis (CIP, N = 1772). [file ACPS-154-142-s001.docx]

| **Online supplement table 1. Risk of death and associated background variables in a sample of individuals with first-episode psychosis and cannabis use disorder (FEP&CUD, N=1360) and cannabis-induced psychosis (CIP, N=1772).** | | | |
| --- | --- | --- | --- |
| **Exposure Variable** | **Hazard Ratio (HR)** | **95% Confidence Interval** | **p-value** |
| FEP&CUD vs. CIP (=ref) | 1.099 | 0.843 - 1.431 | 0.4854 |
|  |  |  |  |
| Gender | 2.204 | 1.342 - 3.622 | 0.0018 |
|  |  |  |  |
| Year of cohort entry | 0.955 | 0.922 - 0.989 | 0.0096 |
|  |  |  |  |
| Age at cohort entry | 1.038 | 1.025 - 1.051 | <.0001 |
|  |  |  |  |
| Sickness absence past year (1-90 days) | 1.065 | 0.680 - 1.669 | 0.7829 |
|  |  |  |  |
| Sickness absence past year (>90 days) | 1.718 | 1.078 - 2.738 | 0.0229 |
|  |  |  |  |
| Disability pension at cohort entry | 1.322 | 0.899 - 1.944 | 0.1566 |
|  |  |  |  |
| Income from work past year | 0.749 | 0.56 - 1.004 | 0.0524 |
|  |  |  |  |

| **Online supplement table 2. Risk of psychosis relapse and associated background variables in a sample of individuals with first-episode psychosis and cannabis use disorder (FEP&CUD, N=1360) and cannabis-induced psychosis (CIP, N=1772).** | | | |
| --- | --- | --- | --- |
| **Exposure Variable** | **Hazard Ratio (HR)** | **95% Confidence Interval** | **p-value** |
| FEP&CUD vs. CIP (=ref) | 1.422 | 1.271 - 1.578 | <.0001 |
|  |  |  |  |
| Gender | 1.217 | 1.048 - 1.413 | 0.0101 |
|  |  |  |  |
| Year of cohort entry | 1.002 | 0.989 - 1.015 | 0.7406 |
|  |  |  |  |
| Age at cohort entry | 0.980 | 0.973 - 0.987 | <.0001 |
|  |  |  |  |
| Sickness absence past year (1-90 days) | 1.027 | 0.87 - 1.212 | 0.7539 |
|  |  |  |  |
| Sickness absence past year (>90 days) | 0.796 | 0.615 - 1.032 | 0.0848 |
|  |  |  |  |
| Disability pension at cohort entry | 0.932 | 0.763 - 1.138 | 0.4895 |
|  |  |  |  |
| Income from work past year | 0.918 | 0.821 - 1.025 | 0.1287 |
|  |  |  |  |
